# Supplementary material for: Periodic spinodal decomposition in double–strengthened medium–entropy alloy
Source: Nat Commun. 2024 Jul 9;15:5757. doi: 10.1038/s41467-024-50078-6 (PMC11233735; doi:10.1038/s41467-024-50078-6)
Supplement: Supplementary file 1 — Supplementary Information [file 41467_2024_50078_MOESM1_ESM.pdf]

SUPPLEMENTARY INFORMATION

Periodic spinodal decomposition in double–strengthened medium–entropy alloy

Hyojin Park[a,b], Farahnaz Haftlang[b,c,d,], Yoon–Uk Heo[c,**], Jae Bok Seol[e], Zhijun Wang[f], Hyoung Seop Kim[b,c,g,h***]*

[a] *Department of Materials Science and Engineering, Pohang University of Science and Technology, Pohang, 37673, Republic of Korea*

[b] *Center for Heterogenic Metal Additive Manufacturing, Pohang University of Science and Technology (POSTECH), Pohang 37673, Republic of Korea*

[c] *Graduate Institute of Ferrous Technology, Pohang University of Science and Technology, Pohang, 37673, Republic of Korea*

[d] *Department of Materials Science & Engineering, Northwestern University, Evanston, IL, 60208, USA*

[e] *Department of Materials Engineering and Convergence Technology, Center for K–Metal & Microscopy, Gyeongsang National University, Jinju 52828, South Korea*

[f] *State Key Laboratory of Solidification Processing, Northwestern Polytechnical University, Xi'an 710072, China*

[g] *Advanced Institute for Materials Research (WPI–AIMR), Tohoku University, Sendai 980–8577, Japan*

[h] *Institute for Convergence Research and Education in Advanced Technology, Yonsei University, Seoul, 03722, Republic of Korea*

*Corresponding author. Farahanz Haftlang

E–mail address: farahnaz.haftlang@gmail.com

**Corresponding author. Yoon–Uk Heo

E–mail address: yunuk01@postech.ac.kr

***Corresponding author. Hyoung Seop Kim

E–mail address: hskim@postech.ac.kr

Supplementary Figures and Tables:

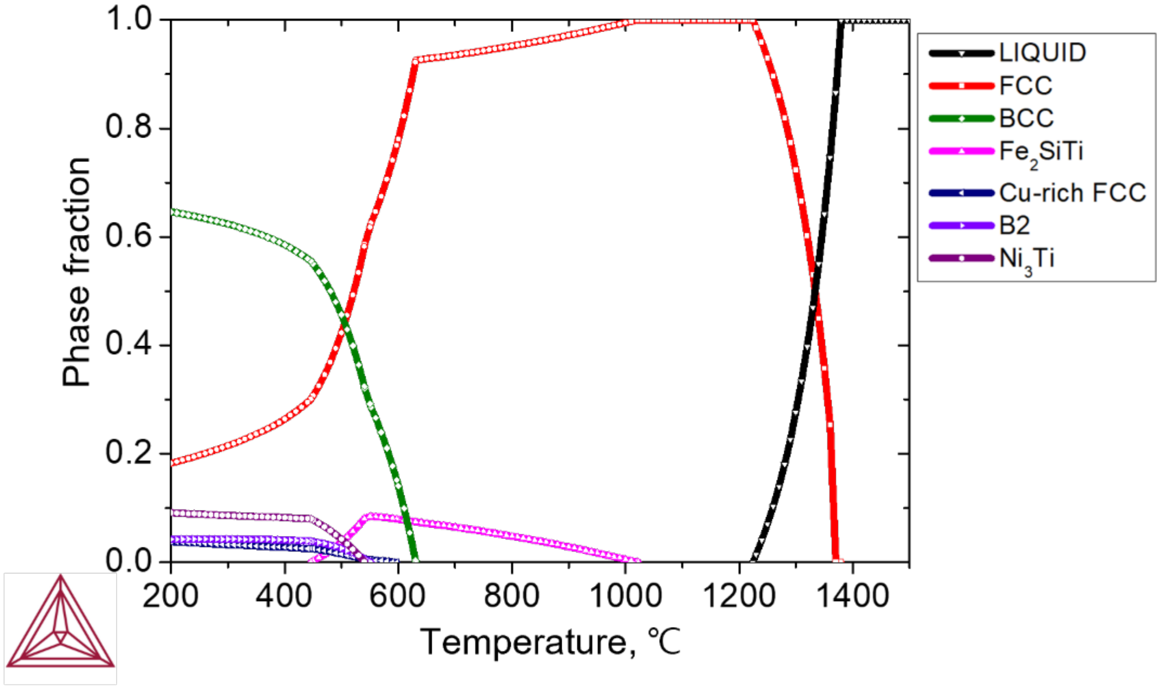

Supplementary Figure 1. Phase diagram of our alloy. Equilibrium phase diagram of the alloy plotting the phase fraction versus temperature.

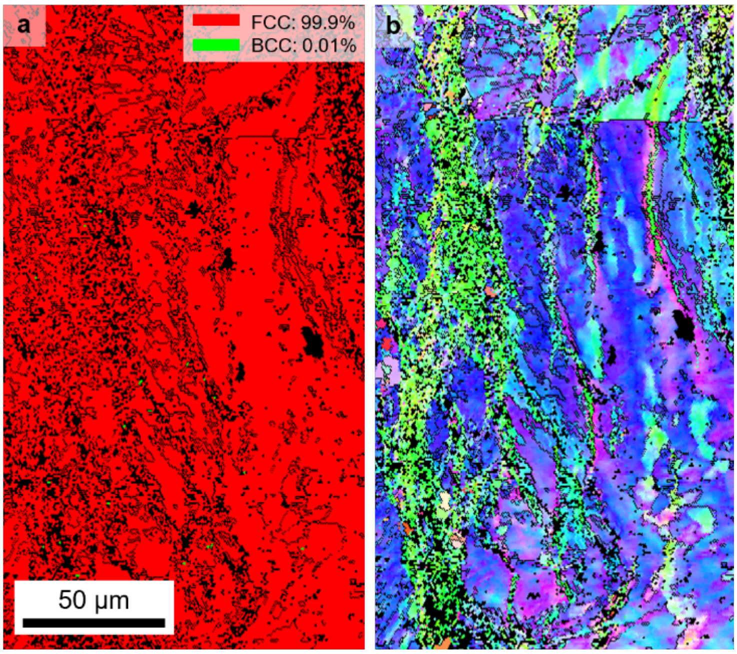

Supplementary Figure 2. **Aged sample.** EBSD **a** phase and **b** IPF maps of the aged sample.

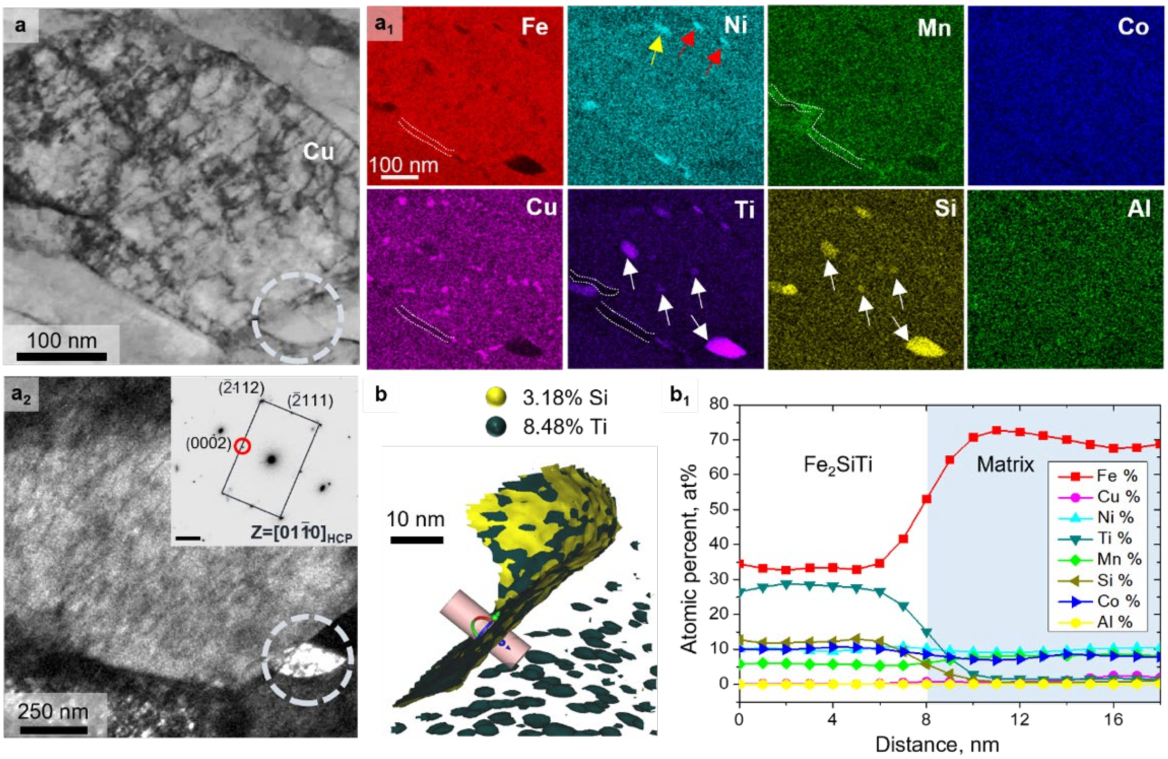

Supplementary Figure 3. Compositional heterogeneity and precipitation of the aged sample. **a** STEM image; **a**$_1$ corresponding EDS maps; **a**$_2$ TEM dark–field image at the exact location as obtained from the red circle in the diffraction pattern in **a**$_2$; **b** 3D APT image reconstructed using isosurface concentrations of 8.48% Ti and 3.18% Si; **b**$_1$ one–dimensional concentration profile across the Fe_2SiTi precipitate along the cylinder in **b**.

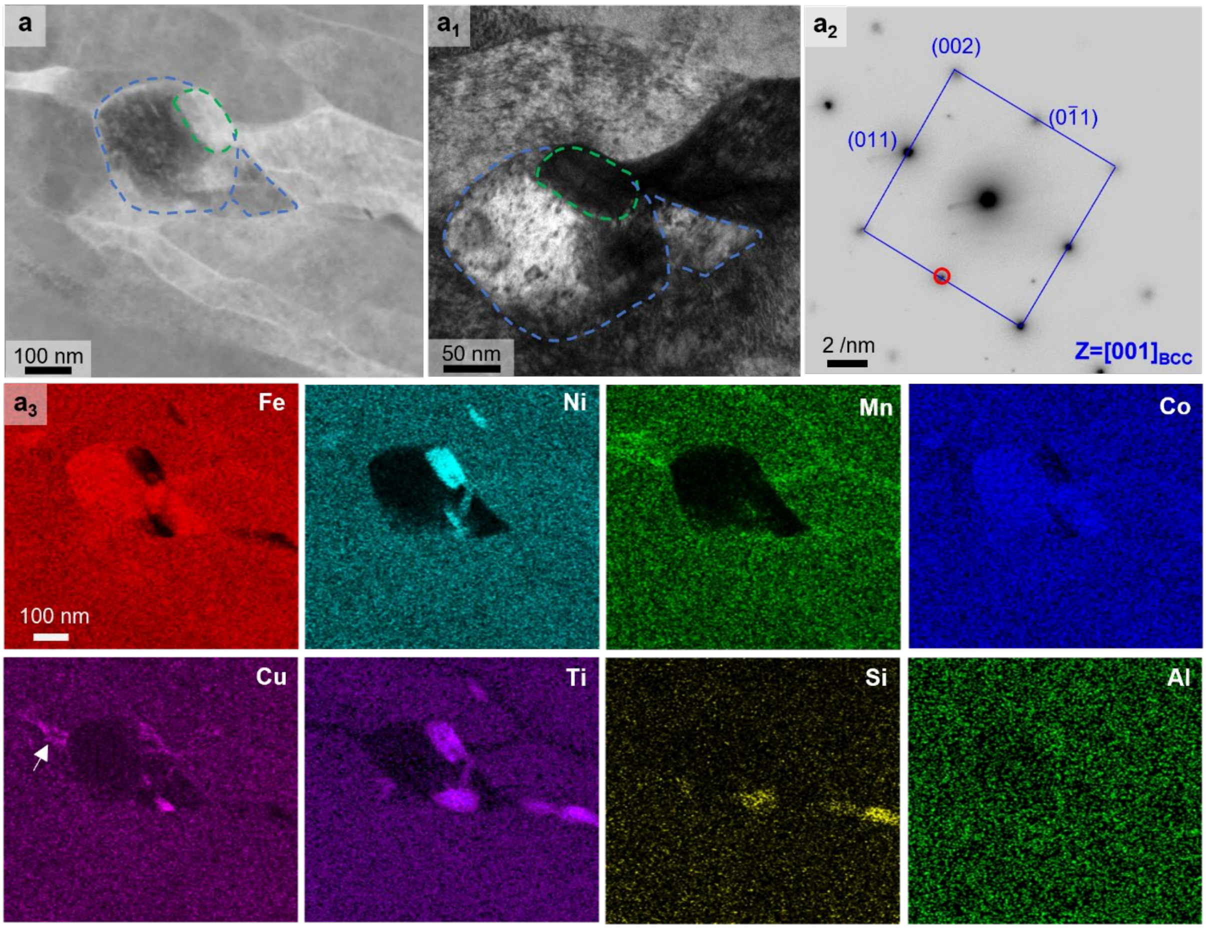

Supplementary Figure 4. Compositional heterogeneity and precipitation for the aged sample. **a** STEM; **a**$_1$ dark–field TEM image obtained from the red circle in **a**$_2$; **a**$_2$ diffraction pattern of BCC indicated by the blue dashed line in **a** and **a**$_1$; **a**$_3$ EDS maps.

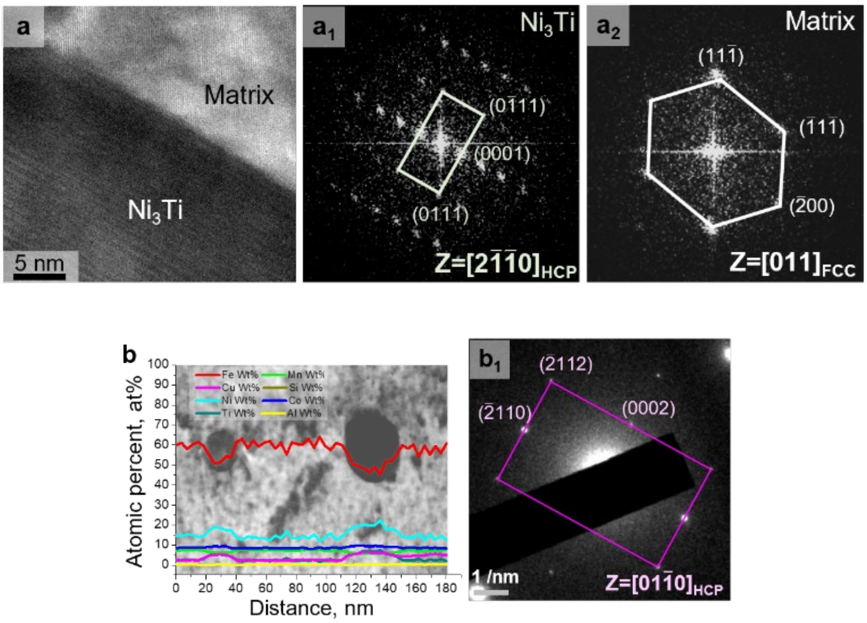

Supplementary Figure 5. Precipitate structure. a High–resolution TEM image of the matrix with Ni_3Ti nanoprecipitation and FFT images of the **a1** Ni_3Ti nanoprecipitate and **a2** matrix. **b** Line scan of the $Ni_3(Ti, Si)_2$ nanoprecipitate. **b1** SAED pattern of the $Ni_3(Ti, Si)_2$ nanoprecipitate.

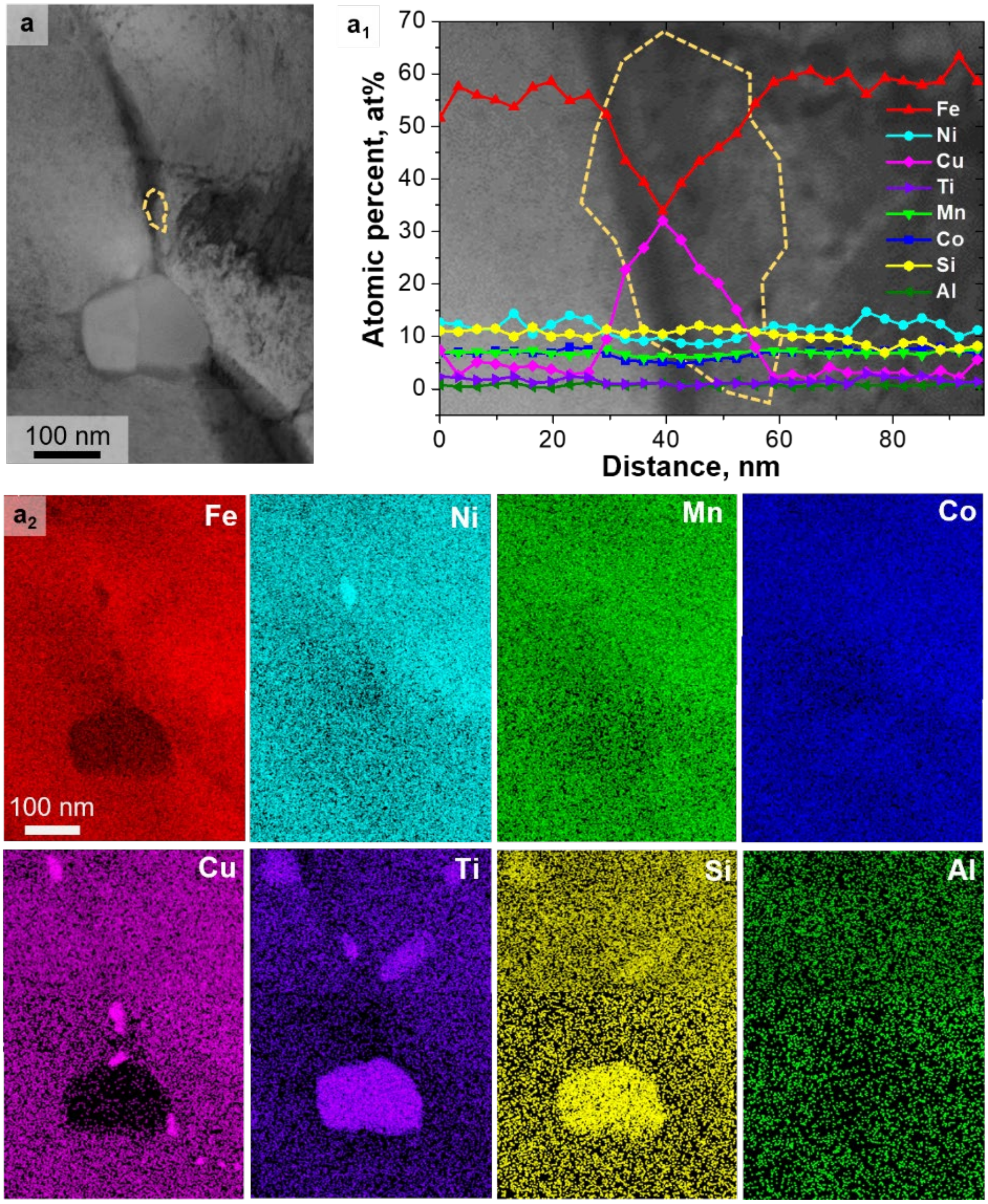

Supplementary Figure 6. Cu cluster. **a** STEM image of the aged sample with the Cu cluster indicated by a yellow dashed line and Fe_2SiTi precipitate; **a**$_1$ line scan of the Cu cluster; **a**$_2$ corresponding EDS maps. The Cu cluster had ~32% Cu enrichment and ~32% Fe depletion, attributed to Cu–Fe immiscibility at intermediate temperatures.

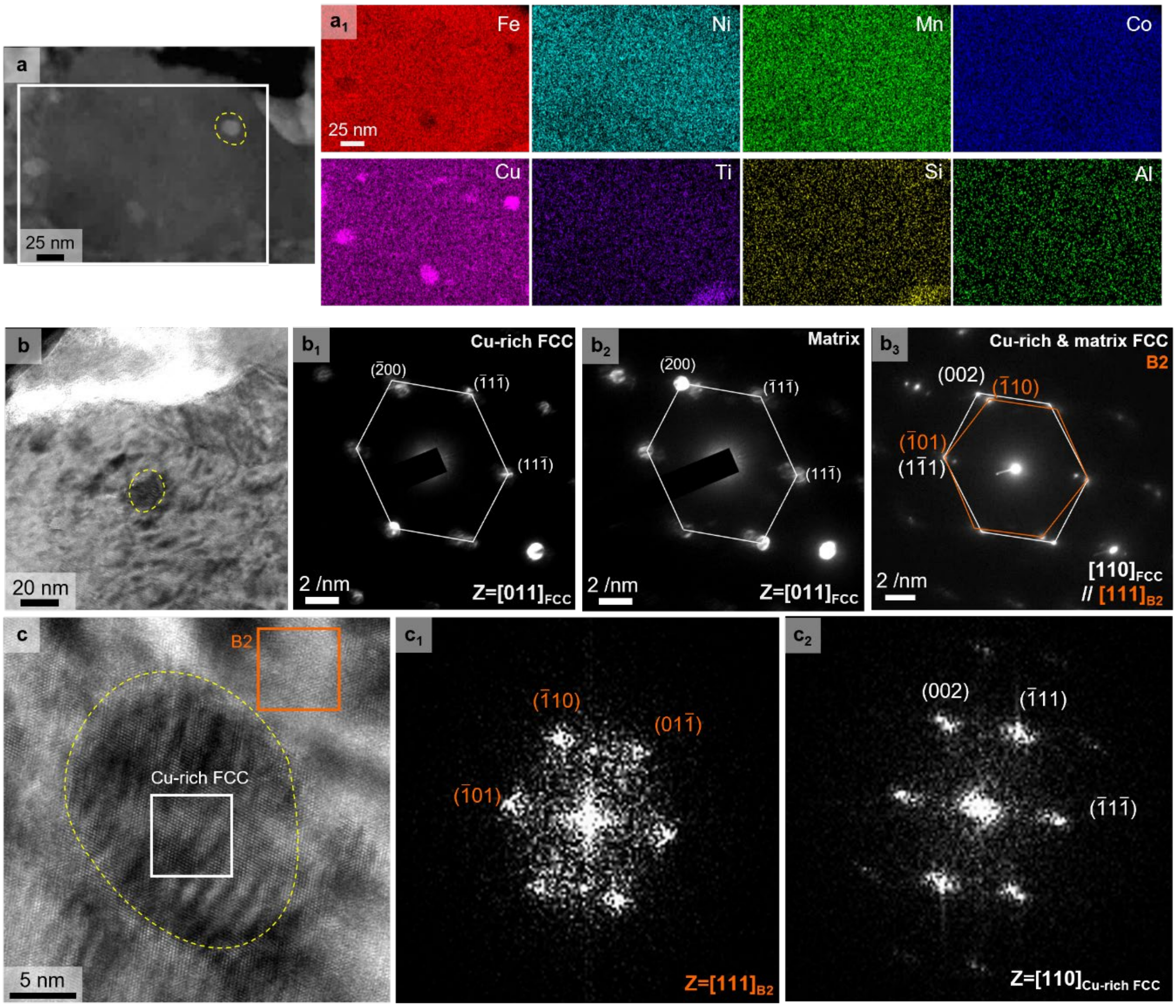

Supplementary Figure 7. B2 structure. a STEM image of Cu–rich FCC precipitate indicated by the yellow line, which is in the same position as **b**–**c**; **a**$_1$ corresponding EDS maps obtained from the white box area of **a.** TEM **b** bright–field image of Cu–rich FCC; nanobeam diffraction of **b**$_1$ Cu–rich FCC and **b**$_2$ FCC matrix; **b**$_3$ SAED pattern. **c** HRTEM image and **c**$_{1–2}$ FFT images of B2 and FCC, respectively.

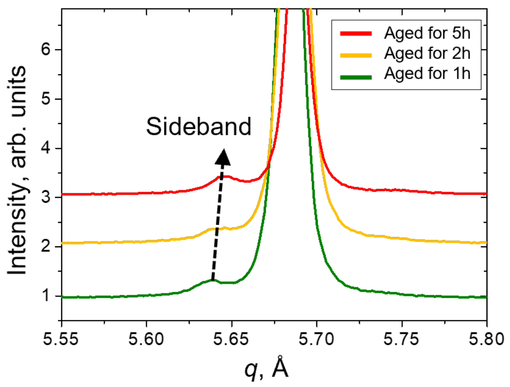

Supplementary Figure 8. Evidence of spinodal decomposition. Synchrotron X–ray {111} diffraction peak and sideband peak of the aged sample at various aging times showing the growth of the sideband peak, i.e., $q = 4\pi\sin\theta/\lambda$, where θ represents the half–scattering angle between the incident beam and scattered beam, and λ represents the wavelength of the incident X–ray.

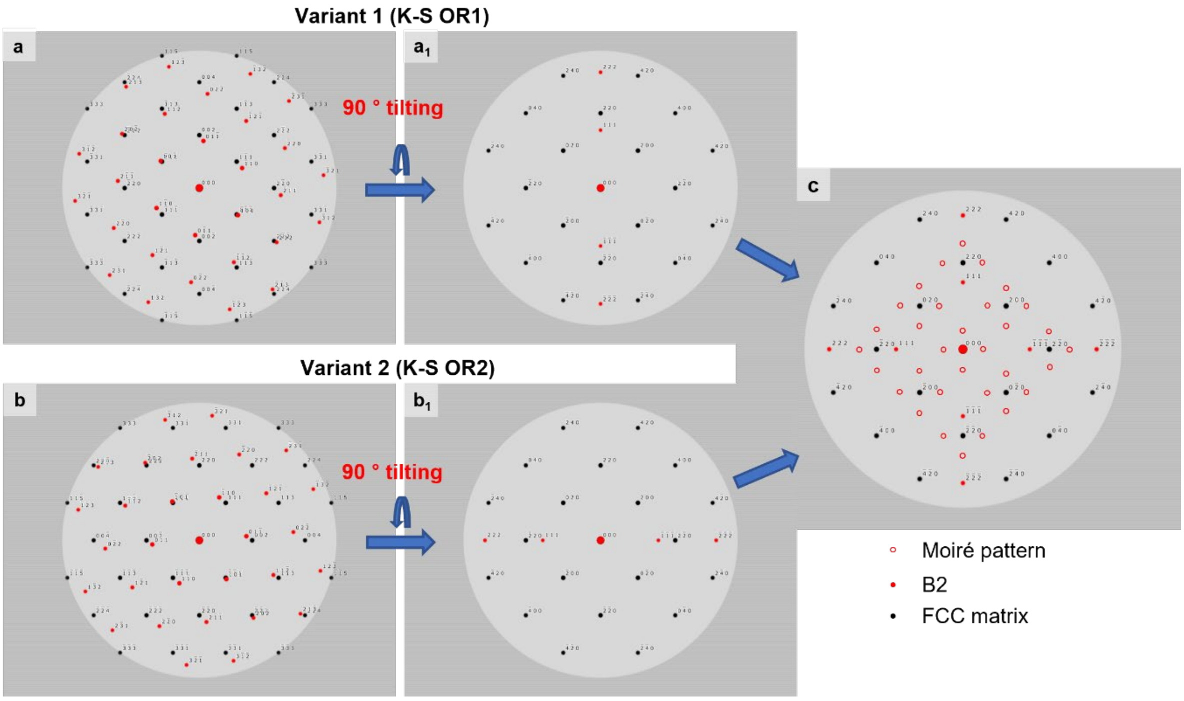

Supplementary Figure 9. Superimposition of FCC and B2 structures. **a** Schematic of variant 1 of the Kurdjumov–Sachs orientation relationship (K–S OR1) and **a**$_1$ schematic of **a** tilted 90°. **b** Schematic of variant 2 of the Kurdjumov–Sachs orientation relationship (K–S OR2) and **b**$_1$ schematic of **b** tilted 90°. Only the (111) plane was indexed to an ordered BCC structure. **c** Image combining the patterns of **a**$_1$ and **b**$_1$ and containing a moiré pattern.

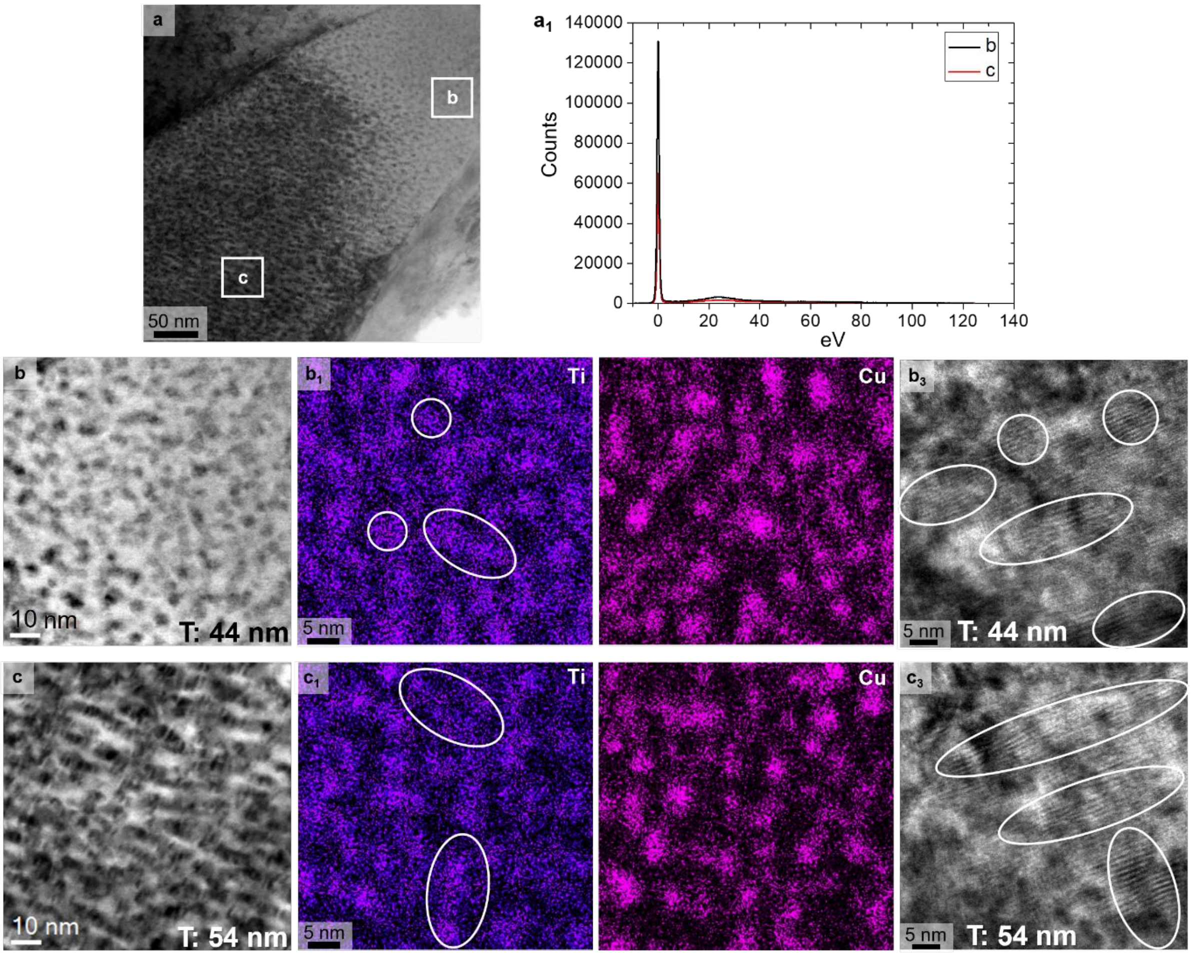

Supplementary Figure 10. Specimen thickness–dependent Moiré patterns. **a** STEM bright–field image showing variation in specimen thickness; **a_1** EELS low–loss spectra corresponding the positions b and c in a. **b** high magnification image of the position b in a; **b_1** EDS maps of Ti and Cu; **b_2** HR image of the corresponding region. **c** high magnification image of the position c in a; **c_1** EDS maps of Ti and Cu; **c_2** HR image of the corresponding region.

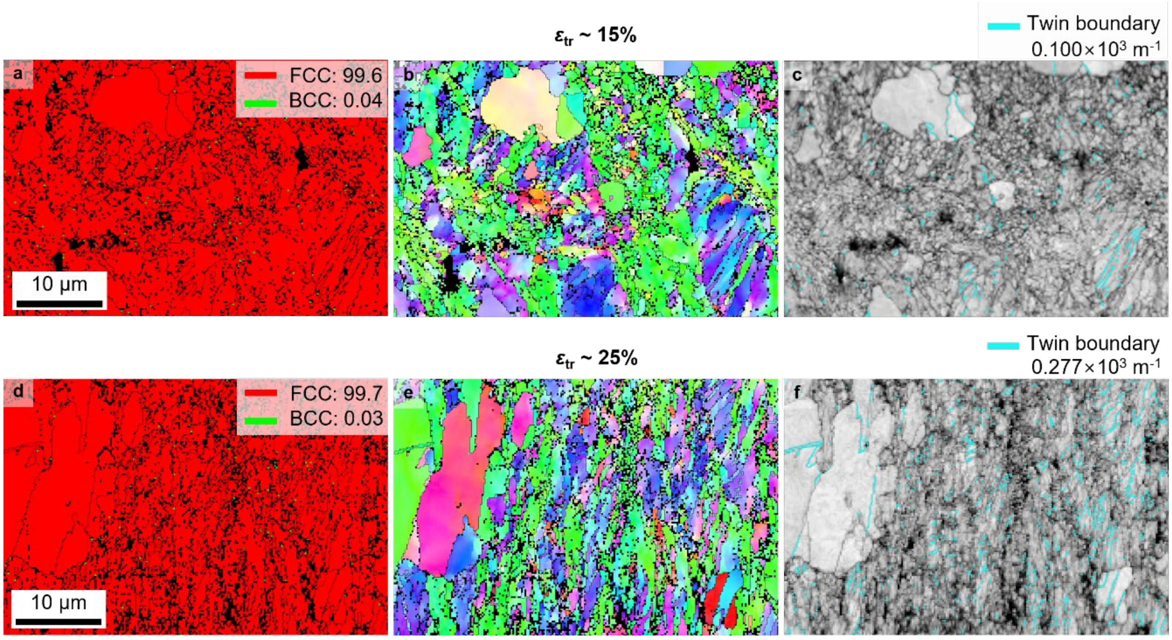

Supplementary Figure 11. **Deformed microstructure.** EBSD **a** phase, **b** IPF and **c** IQ map indexed twin boundaries of the deformed aged samples at 15% of true local strain, and **d** phase, **e** IPF and **f** IQ map indexed twin boundaries of the deformed aged samples at 25% of true local strain.

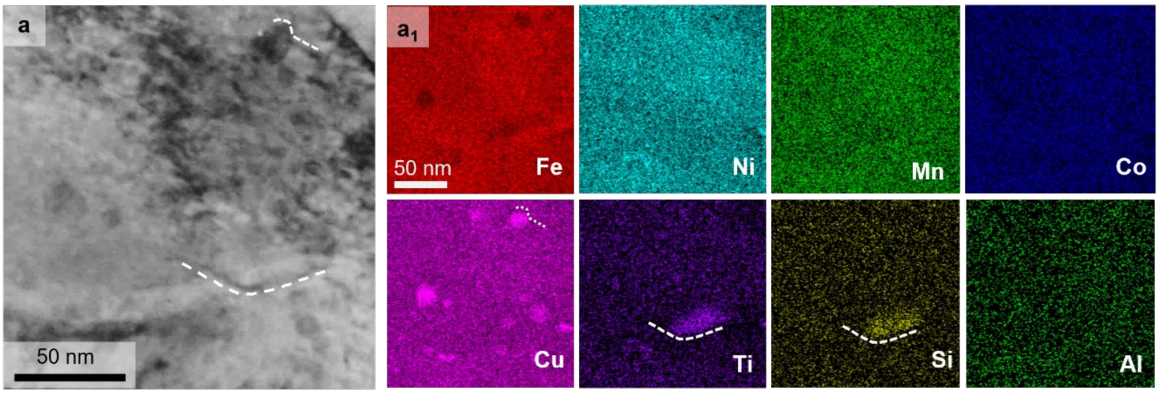

Supplementary Figure 12. Dislocation bowing to the precipitate. a STEM bright–field image and **a**$_1$ corresponding EDS maps.

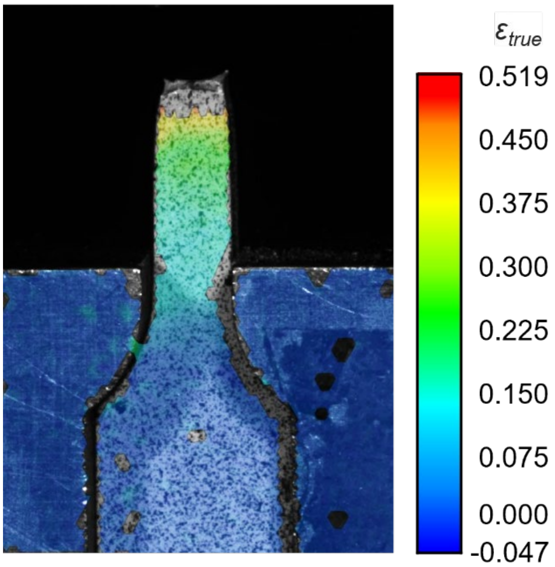

Supplementary Figure 13. DIC image of local true strain distribution. The distribution of local true strain in the aged sample following fracture, as captured by the DIC technique.

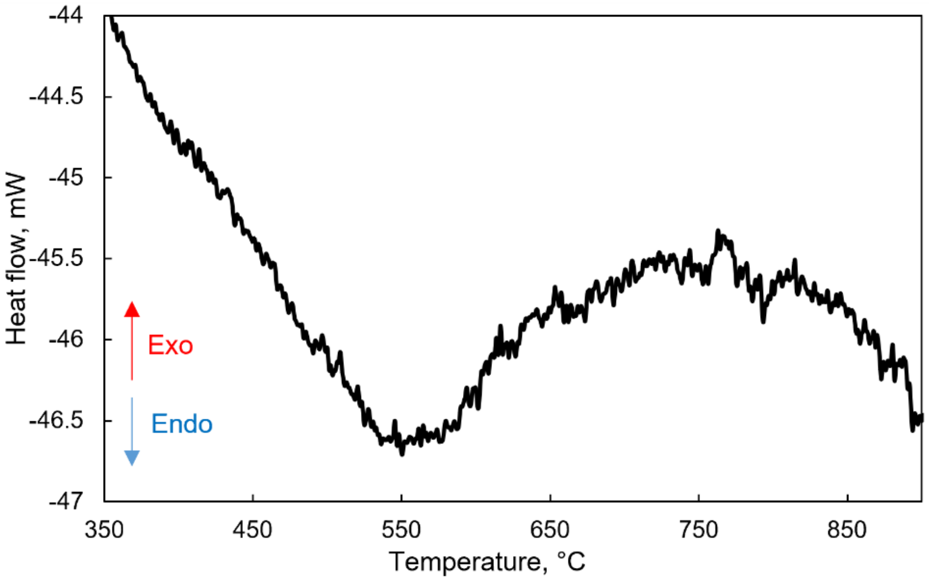

Supplementary Figure 14. DSC curve of the based alloy. Differential scanning calorimetry (DSC) heating curve of our base alloy, $Fe_{65}Ni_{15}Mn_{8}Co_{8}Ti_{3}Si_{1}$. Cold rolled samples were used for DSC analysis with a SETARAM LABSYS Evo DTA/DSC at a heating rate of 30 °C min^{-1} in an Ar gas atmosphere.

Supplementary Video 1. Reconstructed APT video of each element. APT volumes of Cu, Ni, and Ti reconstructed using an isosurface were 7.9%, 11.14%, and 8.48%, respectively.

Supplementary Table 1. Enthalpies of mixing (ΔH_{AB}^{mix} in kJ/mol) of the binary systems (equiatomic composition)[1].

	Fe	Ni	Co	Mn	Cu	Ti	Si	Al
Fe	Fe	–2	–1	0	13	–11	–35	–11
Ni		Ni	0	–8	4	–35	–40	–22
Co			Co	–5	6	–28	–38	–19
Mn				Mn	4	–8	–45	–19
Cu					Cu	–9	–19	–1
Ti						Ti	–66	–30
Si							Si	–19
Al								Al

Supplementary Table 2. Frequency distribution analysis result, obtained from the APT binomial fitting of all substitutional elements confirms strong non–random distributions of Fe, Ni, Ti, Si, and Cu. n_d is the number of degree of freedom for a given ion. The values of the normalized homogenization parameter μ for non–random distribution of the elements are close to 1.

Element	Reduced X^2	n_d	μ
Fe	9,824.903	69	0.9854
Ni	4,514.915	39	0.9472
**Co**	3.554	38	0.0815
Mn	28.594	38	0.2261
Ti	33,223.403	24	0.9876
Si	3,588.829	65	0.9594
Cu	33,424.329	21	0.9859
Al	22.163	4	0.0661

Supplementary Note 1. Alloy design strategy

The design of this alloy strategically exploits the diversity of constituent elements and their enthalpy contributions to promote spinodal decomposition. The thermodynamic principle, $\Delta G = \Delta H - T\Delta S$, where ΔG represents Gibbs free energy, ΔH is the mixing enthalpy, ΔS is the mixing entropy, and T denotes temperature, describes the significance of the high entropy value in M/HEAs. This high entropy stabilizes the Gibbs free energy, promoting a solid solution state in M/HEAs. Contrary to the beneficial "high entropy effect," an increased number of constituent elements can elevate the enthalpy, thereby enhancing the likelihood of spinodal decomposition[2].

The current $Fe_{61.75}Ni_{14.25}Co_{7.6}Mn_{7.6}Ti_{2.85}Si_{0.95}Cu_{4.5}Al_{0.5}$ MEA alloy has been designed as a branch of Fe-Ti-Si-based nanoprecipitation-strengthened medium-entropy alloys inspired by the previous alloy $Fe_{65}Ni_{15}Mn_{8}Co_{8}Ti_{3}Si_{1}$[3] and $Fe_{68}Ni_{10}Mn_{10}Co_{10}Ti_{1.5}Si_{0.5}$[4]. An extraordinarily high mechanical strength has been reported after the formation of Fe_2SiTi and Ni_3Ti nanoprecipitates, yet with high ductility resulting from the presence of non–shearable precipitates[3-11]. On the other hand, the strong ordering tendencies of Al, along with most of the other alloying elements, make the ordering transition of primary importance. A sufficient concentration of Al encouraged a series of phase transformations, including the formation of coherent mixtures of B2 and partially ordered aluminides in a body–centered cubic (BCC) solid–solution matrix[12-14]. In the presence of Al–containing B2 or BCC phase, the mechanical strength incredibly increases at the expense of ductility. Therefore, to avoid these phase transformations leading to ductility reduction, while using the advantage of lattice expansion and solid solution strengthening, a low concentration of 0.5% was chosen for the Al element. The Fe–Cu binary system is widely known for its positive mixing enthalpy, which significantly tends towards phase separation. In the Fe–based alloy and CrMnFeCoNi alloy systems,

previous literature has revealed microstructural changes with the addition of Cu[15-18]. The addition of Cu above 5 at% can induce Cu–rich precipitation or microscale phase separation[18]. Therefore, in our study, we have strategically limited the Cu addition to below 5% to induce the desired nanostructures without promoting excessive phase separation.

By controlling the composition and microstructure through thermal processing associated with the phase transformations, this system provides a wide range of potential compounds and associated microstructures. Thus, the main concept of the present work is to design a stable FCC matrix to enhance its sensitivity to spinodal decomposition. Based on the thermodynamic calculations and the equilibrium phase diagram presented in **Supplementary Figure 1**, the presence of Fe_2SiTi, Ni_3Ti, B2, and Cu–rich FCC phases is possible upon a suitable heat treatment in the range of ~450–600 °C. Therefore, by applying a similar aging treatment as we have done in our previous work[3] (at 550 °C for 5 hours), as shown in the differential scanning calorimetry (DSC) analysis in **Supplementary Figure 14**), all the aforementioned precipitates have been formed in our microstructure.

Supplementary Note 2. Multiple dynamic precipitates in the aged sample

To clarify the microstructural features of the aged sample, TEM and APT analyses were conducted to investigate the nanoscale microstructural evolution during aging. The STEM image and corresponding EDS maps revealed several distinct compositional heterogeneities in the form of precipitates with distinctive elemental distributions, as follows:

(i) The presence of a Fe_2SiTi precipitate with a hexagonal close–packed (HCP) structure (P63/mmc, a = 4.77 Å, c = 7.74 Å) in the Fe–Mn–Cu–depleted area was confirmed by the SAED pattern corresponding to the area indicated by the white dashed line in the STEM and TEM dark–field images (see **Supplementary Figures 3a** and **a_2**). Additionally, the reconstructed APT concentration profile (**Supplementary Figure 3b_1**) of the Fe_2SiTi precipitate in (**Supplementary Figure 3b**) indicated high concentrations of Si and Ti across the precipitate.

(ii) In addition to Fe_2SiTi, as indicated by white arrows in the EDS map in **Supplementary Figure 3a_1**, the presence of Ni– and Ti–rich areas (indicated by red arrows in the Ni EDS map in **Supplementary Figure 3a_1**) suggested the formation of Ni_3Ti nanoprecipitates. A perspective illustration of the Ni_3Ti precipitate is shown by the green dashed line in the STEM and dark–field images in **Supplementary Figures 4a** and **a_1**; the Ni_3Ti precipitate is enriched in Ni and Ti and depleted in Fe, Cu, Mn, and Co, as confirmed by the corresponding EDS results in **Supplementary Figure 4a_3**. The FFT in **Supplementary Figures 5a–a_2** identifies the Ni_3Ti precipitate as an η–$D0_{24}$ nanoprecipitate with an HCP structure (P63/mmc, a = 5.074 Å, and c = 8.31 Å) along the $[2\bar{1}\bar{1}0]_\eta$ zone axis, with the matrix identified as FCC along the [011] zone axis. The Ni_3Ti precipitate exhibits semi–coherency with the FCC matrix in the Nishiyama–Wassermann orientation with $(1\bar{1}1)_{FCC}||(0001)_\eta$ and $[011]_{FCC}||[2\bar{1}\bar{1}0]_\eta$[19]. Ni_3Ti and Fe_2SiTi precipitates were abundant in the alloys, typically with

average sizes of 20–200 nm.

(iii) More interestingly, the $Ni_3(Ti, Si)_2$ nanoprecipitate—indicated by the yellow arrow in the Ni map in **Supplementary Figure 3a$_1$**—was confirmed as HCP (P63/mmc, a = 4.57 Å, and c = 7.96 Å) along the $[01\bar{1}0]_{HCP}$ zone axis, as shown in **Supplementary Figures 5b** and **b$_1$**. $Ni_3(Ti, Si)_2$ is a metastable phase commonly observed in Ni–Ti–based alloys[20,21]. The metastable phase can coarsen and decompose at high aging temperatures with long aging times to form the stable phase Ni_3Ti[21].

(iv) In addition to the precipitates, constitutive elements were segregated near the dislocation lines, confirming the role of special boundaries as the preferential diffusion paths for precipitate growth. The EDS maps in **Supplementary Figure 4a$_3$** reveal the segregation and depletion of Fe, Mn, Cu, Ti, and Co at the grain boundaries surrounding the Ni_3Ti and Fe_2SiTi precipitates. This boundary segregation increases the energy barrier for dislocation motion, thus enhancing the overall material strength[22]. Fe diffused along the dislocation lines and accumulated in the interior regions, creating a region with a locally high Fe concentration, as indicated by the blue dashed line in **Supplementary Figures 4a** and **a$_1$**. Excess Fe in the grain can induce the transformation of the FCC phase into the body–centered cubic (BCC) phase by reducing the FCC phase stability[23]. According to the diffraction pattern (**Supplementary Figure 4a$_2$**), the Fe–rich particles were identified as a new BCC structure formed during aging. The white arrow in **Supplementary Figure 4a$_3$** indicates that Cu was segregated at the grain boundaries as a distinct diffusion channel compared with Fe. Cu clusters were formed owing to the aforementioned Cu segregation, leading to Cu depletion at the grain–boundary regions (**Supplementary Figure 3a$_1$**).

(v) Compared with other constitutive elements, Cu has distinct characteristics because of its segregation at both precipitates and grain boundaries. Instead, as shown in the Cu EDS map in **Supplementary Figure 3a$_1$**, Cu formed isolated clusters approximately 10–20 nm in

size located in the grain interior and in the vicinity of the precipitates. The chemical composition of the Cu clusters was analyzed via line scanning, as shown in **Supplementary Figures 6a–a_2**. The Cu cluster was enriched with approximately 32% Cu, whereas the Fe content was reduced to 32%. This preferential segregation of Cu is attributed to the immiscibility of the Cu–Fe binary system at intermediate temperatures caused by the high positive mixing enthalpy between the two elements.

(vi) Independent of the Cu cluster, STEM and EDS images indicated a Cu–rich FCC precipitate (see **Supplementary Figures 7a** and **a_1**)—confirmed via bright–field imaging and nanobeam diffraction along the [011] axis, as shown in **Supplementary Figures 7b** and **b_1**. The SAED pattern (**Supplementary Figure 7b_3**) obtained from **Supplementary Figure 7b** reveals the superposition of the FCC pattern from the Cu–rich FCC and the matrix and the B2 pattern, which exhibits the Kurdjumov–Sachs orientation relationship. The HRTEM and FFT images in **Supplementary Figures 7c–c_2** provide further insight into the selected areas. The B2 spots existed in a small area next to the Cu–rich FCC. However, no elemental segregation was observed apart from that of the Cu–rich FCC in **Supplementary Figure 7a_1**.

Supplementary References

1 Takeuchi, A. & Inoue, A. Classification of bulk metallic glasses by atomic size difference, heat of mixing and period of constituent elements and its application to characterization of the main alloying element. *Mater. Trans.* **46**, 2817-2829 (2005).

2 Luan, H.W. *et al.* Spinodal decomposition and the pseudo-binary decomposition in high-entropy alloys. *Acta Mater.* **248** (2023).

3 Haftlang, F. *et al.* Simultaneous effects of deformation-induced plasticity and precipitation hardening in metastable non-equiatomic FeNiCoMnTiSi ferrous medium-entropy alloy at room and liquid nitrogen temperatures. *Scr. Mater.* **202** (2021).

4 Haftlang, F., Seol, J.B., Zargaran, A., Moon, J. & Kim, H.S. Chemical core-shell metastability-induced large ductility in medium-entropy maraging and reversion alloys. *Acta Mater.* **256**, 119115 (2023).

5 Fillon, A. *et al.* Influence of severe plastic deformation on the precipitation hardening of a FeSiTi steel. *J. Mater. Sci.* **47**, 7939-7945 (2012).

6 Perrier, M., Bouaziz, O., Brechet, Y., Deschamps, A. & Donnadieu, P. Mechanical properties of low carbon steel hardened by the Fe2SiTi phase at high volume fraction. *J. Phys.: Conf. Ser.* **240,** 012095 (2010).

7 Knowles, A.J. *et al.* Development of Ni-free Mn-stabilised maraging steels using Fe2SiTi precipitates. *Acta Mater.* **174**, 260-270 (2019).

8 Perrier, M. *et al.* Precipitation sequence and kinetics in an Fe-Si-Ti alloy. *Solid State Phenom.* **172**, 833-838 (2011).

9 Perrier, M. *et al.* Characterization and modeling of precipitation kinetics in a Fe-Si-Ti alloy. *Metall. Mater. Trans. A* **43**, 4999-5008 (2012).

10 Löffler, F., Palm, M. & Sauthoff, G. Iron-Rich Iron-Titanium-Silicon Alloys with Strengthening Intermetallic Laves Phase Precipitates. *Steel Res. Int.* **75**, 766-772 (2004).

11 Jack, D. & Honeycombe, R. Age hardening of an Fe-Ti-Si alloy. *Acta Metall.* **20**, 787-796 (1972).

12 Santodonato, L.J. *et al.* Deviation from high-entropy configurations in the atomic distributions of a multi-principal-element alloy. *Nature communications* **6**, 5964 (2015).

13 Singh, S., Wanderka, N., Murty, B., Glatzel, U. & Banhart, J. Decomposition in multi-component AlCoCrCuFeNi high-entropy alloy. *Acta Mater.* **59**, 182-190 (2011).

14 Santodonato, L.J., Liaw, P.K., Unocic, R.R., Bei, H. & Morris, J.R. Predictive multiphase evolution in Al-containing high-entropy alloys. *Nat. commun* **9**, 4520 (2018).

15 Ma, X. *et al.* Spinodal decomposition of precipitation hardening Fe-17Cr-4Ni-4Cu stainless steel at 475 °C. *Materials and Technology* **56**, 193–199-193–199 (2022).

16 Fiocchi, J., Mostaed, A., Coduri, M., Tuissi, A. & Casati, R. Development and characterization of a novel high entropy alloy strengthened through concurrent spinodal decomposition and precipitation. *J. Alloy. Compd.* **947**, 169706 (2023).

17 Zhang, Y. *et al.* Concurrence of spinodal decomposition and nano-phase precipitation in a multi-component AlCoCrCuFeNi high-entropy alloy. *J. Mater. Res. Technol* **8**, 726-736 (2019).

18 Du, C. *et al.* Effect of Cu on the strengthening and embrittling of an FeCoNiCr-xCu HEA. *Mater. Sci. Eng. A* **832**, 142413 (2022).

19 Suk, J.I., Hong, S.H. & Nam, S.W. Crystallographic Orientation Relationships among Eta-Ni3-Ti Precipitate, Reverted Austenite, and Martensitic Matrix in Fe-10cr-10ni-2w Maraging Alloy. *Metall. Mater. Trans. A* **24**, 2643-2652 (1993).

20 Chen, H., Zheng, L.J., Zhang, F.X. & Zhang, H. Thermal stability and hardening behavior in superelastic Ni-rich Nitinol alloys with Al addition. *Mat. Sci. Eng. A* **708**,

514-522 (2017).

21 Nishida, M., Wayman, C.M. Electron microscopy studies of precipitation processes in near-equiatomic TiNi shape memory alloys. *Mater.Sci. Eng.* **93**, 191-203 (1987).

22 Raabe, D. *et al.* Grain boundary segregation engineering in metallic alloys: A pathway to the design of interfaces. *Curr. Opin. Solid State Mat. Sci.* **18**, 253-261 (2014).

23 Haftlang, F., Kim, E.S. Kwon, J., Heo, Y.-U., Kim, H.S. Extraordinary combination of strength and ductility in an additively manufactured Fe-based medium entropy alloy through in situ formed η-nanoprecipitate and heterogeneous microstructure. *Addit. Manuf.* **63**, 103421 (2023).
