# Supplementary material for: Periodic spinodal decomposition in double–strengthened medium–entropy alloy
Source: Nat Commun. 2024 Jul 9;15:5757. doi: 10.1038/s41467-024-50078-6 (PMC11233735; doi:10.1038/s41467-024-50078-6)
Supplement: Supplementary file 3 — Description of Additional Supplementary Files [file 41467_2024_50078_MOESM3_ESM.pdf]

Supplementary Video 1. Reconstructed APT video of each element. APT volumes of Cu, Ni, and Ti reconstructed using an isosurface were 7.9%, 11.14%, and 8.48%, respectively.
